# Examination of endoscopic intracanal observation with an ultrafine‐diameter scope

**DOI:** 10.1002/deo2.70053

**Published:** 2025-01-06

**Authors:** Takeshi Jinno, Kazuya Miyaguchi, Daiki Muraishi, Toshiyuki Narumi, Kanji Kabasawa, Hideki Ohgo, Yoshikazu Tsuzuki, Hiroyuki Imaeda

**Affiliations:** ^1^ Department of Clinical Engineering Saitama Medical University Saitama Japan; ^2^ Department of Gastroenterology Saitama Medical University Saitama Japan

**Keywords:** endoscope inspection, endoscopic intracanal injury, EYEBOSCO, SDGs, ultrafine scope

## Abstract

**Objectives:**

The endoscopic channel can be damaged by instruments during use and cleaning, leading to contamination, infection, and increased repair costs. However, few devices are available to observe the inside of the endoscopic channel. This study employed an ultrafine‐diameter scope to examine damage in the endoscopic channel.

**Methods:**

Fifty‐eight endoscopes used at our institution were examined for scratches, discoloration, or deformation in the endoscopic channel using an ultrafine‐diameter scope.

**Results:**

Damage was observed in seven of the 24 observation endoscopes and 27 of the 34 therapeutic endoscopes, with damage being more common in the therapeutic endoscopes. Scratches were observed in nine of the 25 upper gastrointestinal endoscopes, 23 of the 24 colonoscopes, and one of the two echoendoscopes. Additionally, two colonoscopes, one echoendoscope, and one double‐balloon endoscope showed indentation or narrowing near the curvature.

**Conclusions:**

The use of an ultrafine‐diameter scope enabled the detection of minute damage and deformations in the channel. Periodic observation with the ultrafine‐diameter scope may promote the long‐term use of the scopes.

## INTRODUCTION

The inside of the endoscopic channel can be damaged during use or by cleaning tools. Such damage can lead to issues, including contamination, infection, and costly repairs due to scope malfunction, as well as difficulties in cleaning and disinfection caused by the complicated scope structure.^1^ European guidelines recommend that endoscopes be disinfected immediately before the first patient examination of the day and during periodic bacterial tests of gastrointestinal endoscopic equipment.^2^ Infections following gastrointestinal endoscopy are most frequently caused by the patient's endogenous intestinal microbiota.

A systematic review of 117 articles revealed that the composite infection rate was 0.2% following gastrointestinal endoscopic procedures, 0.8% following endoscopic retrograde cholangiopancreatography (ERCP), 0.123% following non‐ERCP upper gastrointestinal endoscopic procedures, and 0.073% following lower gastrointestinal endoscopic procedures.^3^

In Japan, periodic culture tests and other cleanliness assessments are considered desirable to ensure the hygienic quality of endoscopic devices. However, no reports exist on devices specifically designed to observe the inside of the channels. Recently, green endoscopy has gained prominence for its potential to reduce waste, conserve water and energy resources, and minimize unnecessary endoscopic examinations.^4^

Treating endoscopes with care is crucial, and this includes performing periodic endoscope inspections. In this report, we discuss our observations of the inside of an endoscopic channel using the EYEBOSCO (ABIS Inc, Hyogo, Japan), an ultrafine‐diameter scope.

## METHODS

### Study design

**FIGURE 1:**
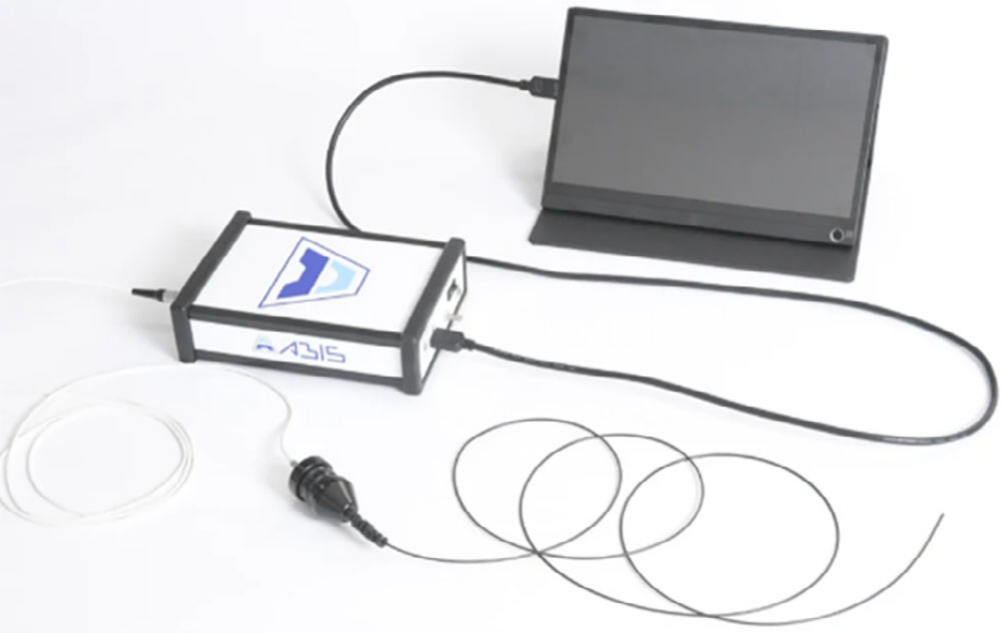
Appearance of EYEBOSCO.

**FIGURE 2:**
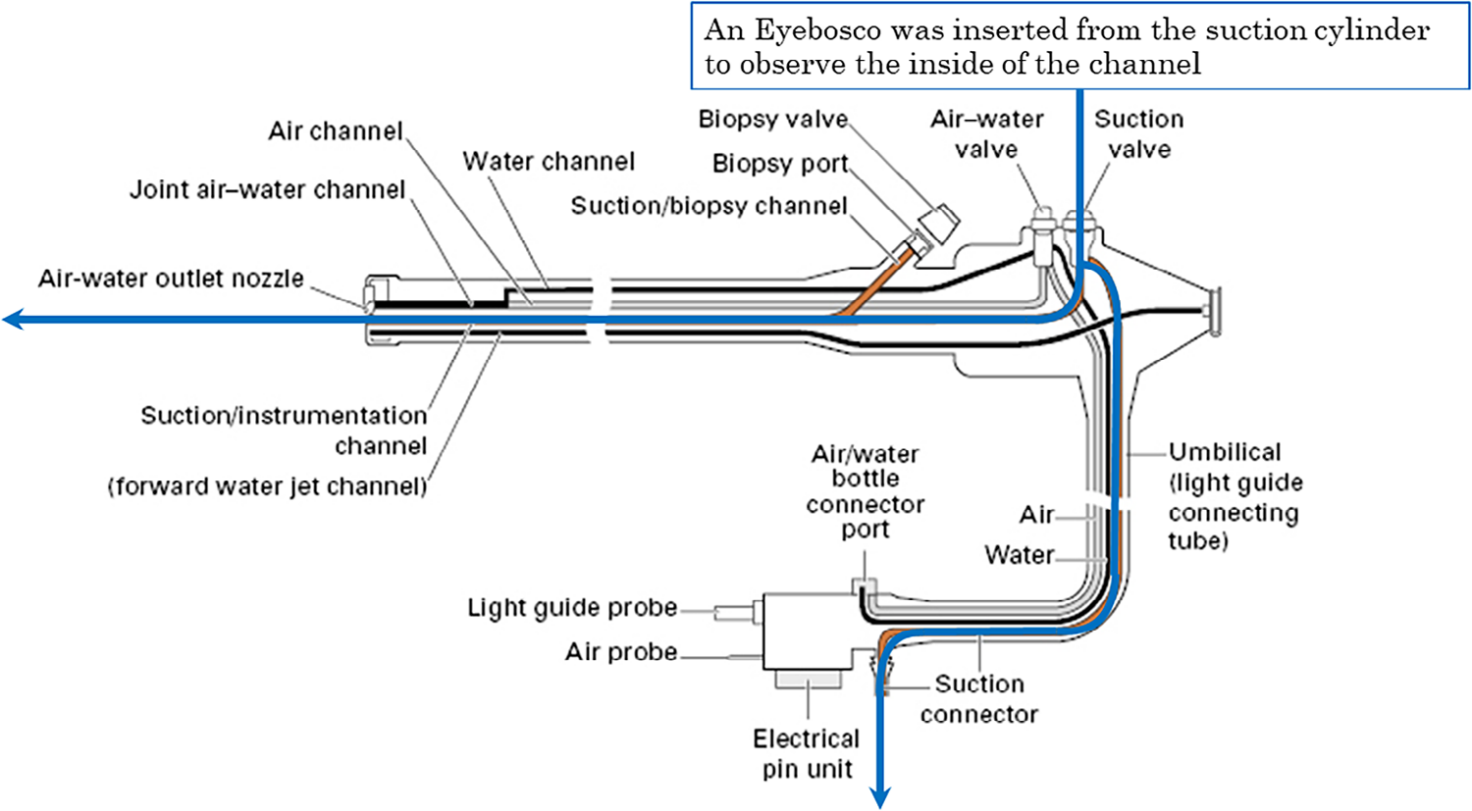
Lumen structure of the scope (blue arrows indicate areas observed in this study). Retrieved and modified from https://abdominalkey.com/2‐endoscopic‐equipment.

The EYEBOSCO was inserted through the suction cylinder of the endoscope for observation and video recording (Figure 1). The internal structure of the endoscope is shown in Figure 2. EYEBOSCO is a reusable product and requires cleanliness treatment after use. Therefore, it was disinfected with an alcohol cleaning cloth following the observation. The endoscope was also disinfected using EYEBOSCO after high‐level disinfection and again after use. The observation paths were as follows: from the suction cylinder to the forceps port at the end of the scope and from the suction cylinder to the suction port. The video recordings were examined for any flaws, discoloration, and channel deformation.

### Definition of injuries

**FIGURE 3:**
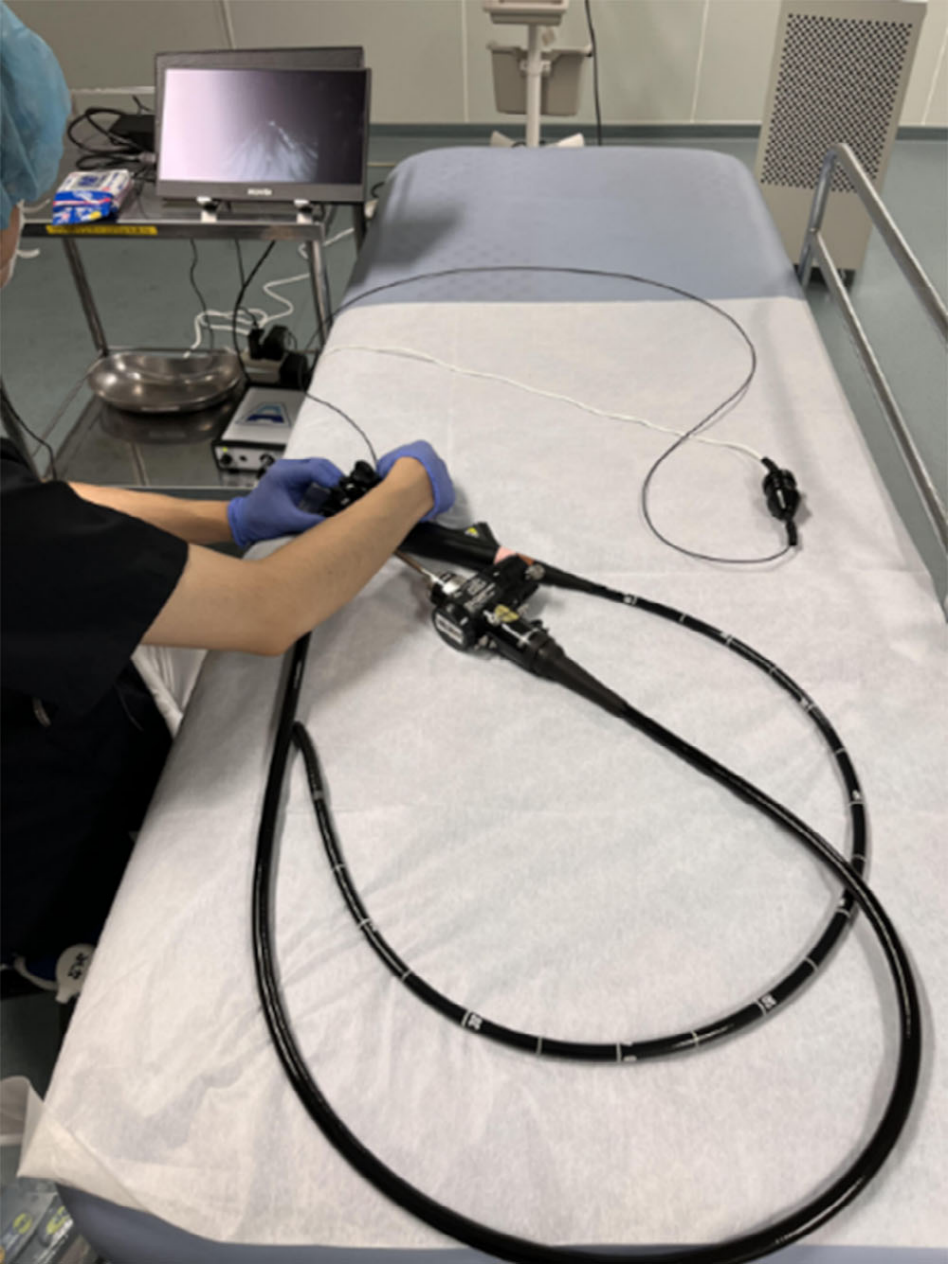
Actual observation of the lumen of an endoscope using EYEBOSCO.

**FIGURE 4:**
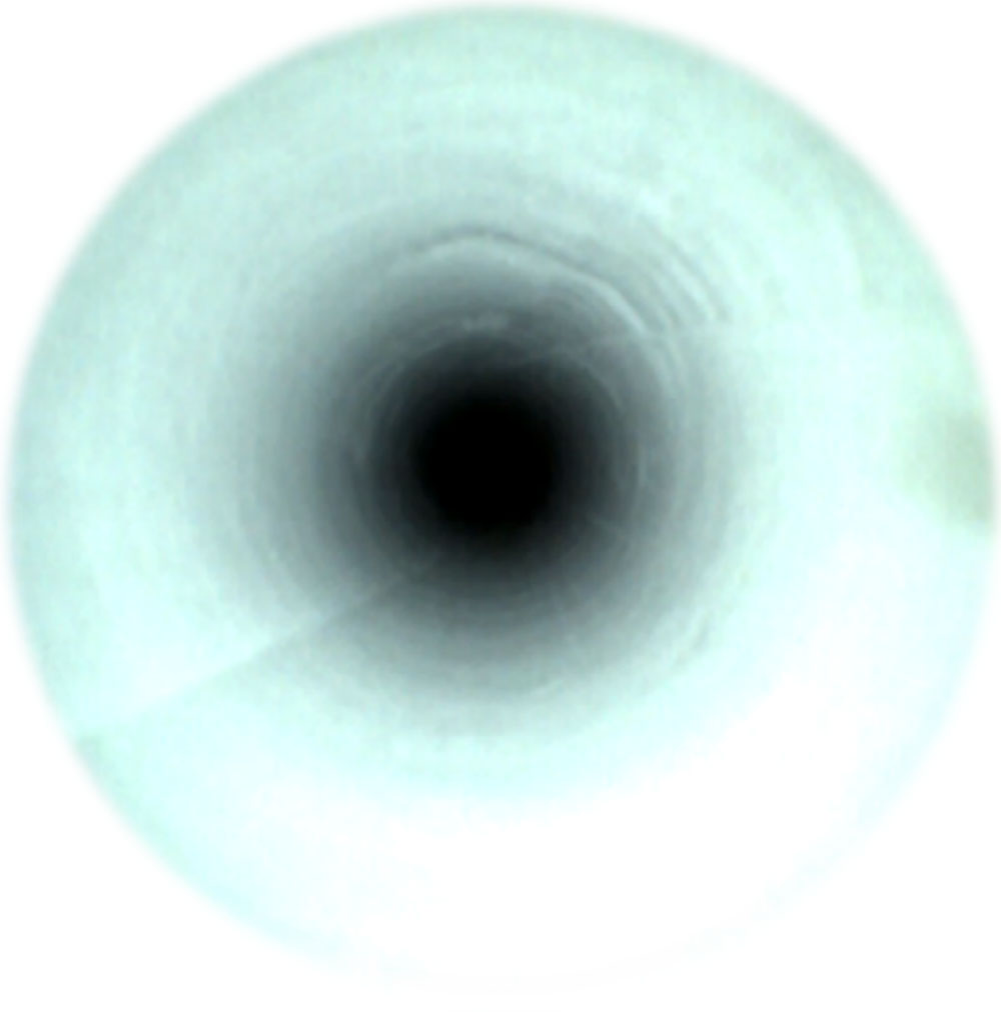
Normal image of the endoscopic channel.

**FIGURE 5:**
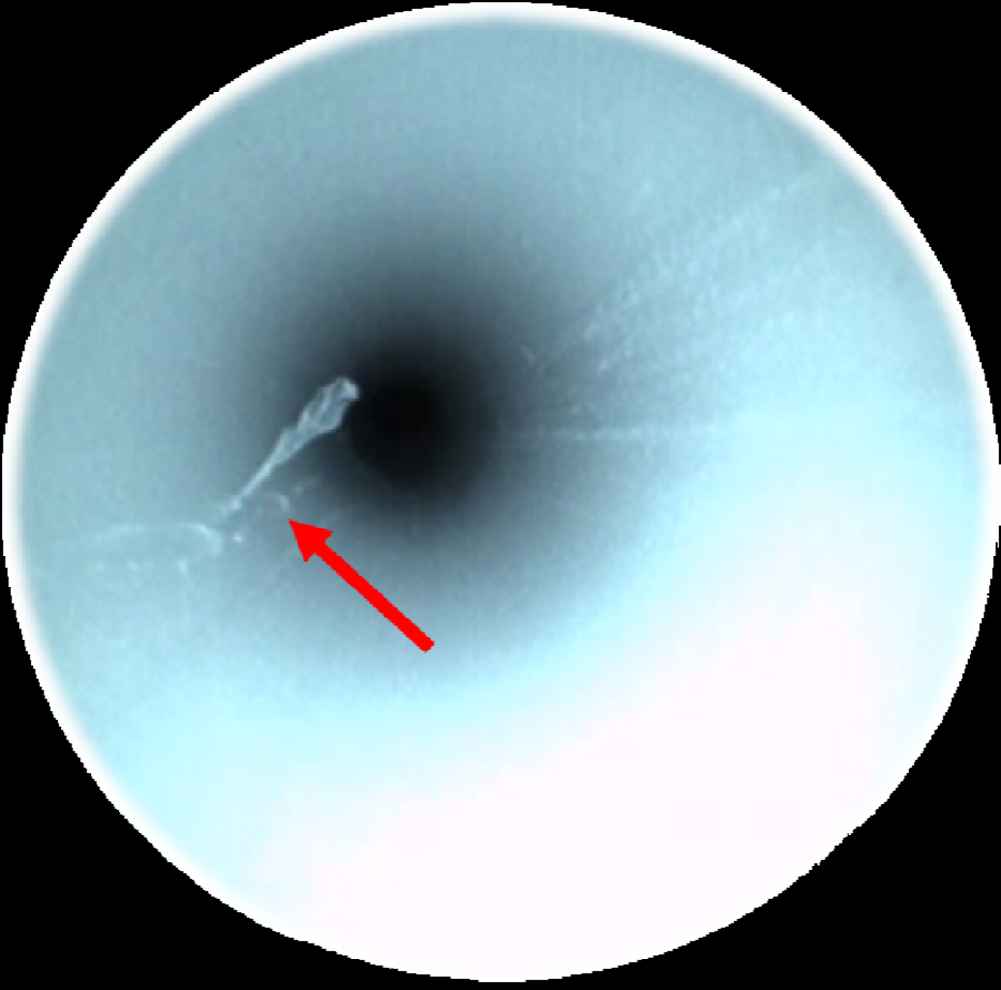
Scratches in the endoscopic channel (indicated by red arrows).

**FIGURE 6:**
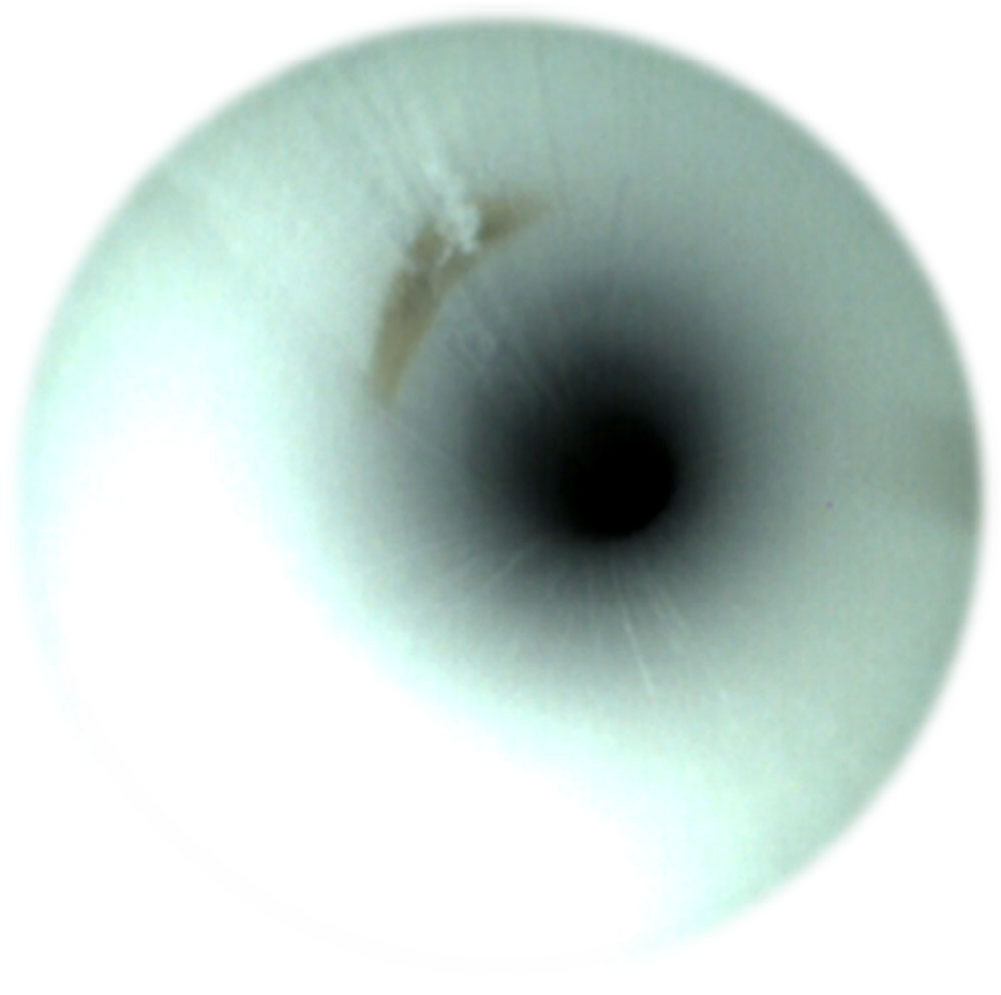
Discoloration in the endoscopic channel.

**FIGURE 7:**
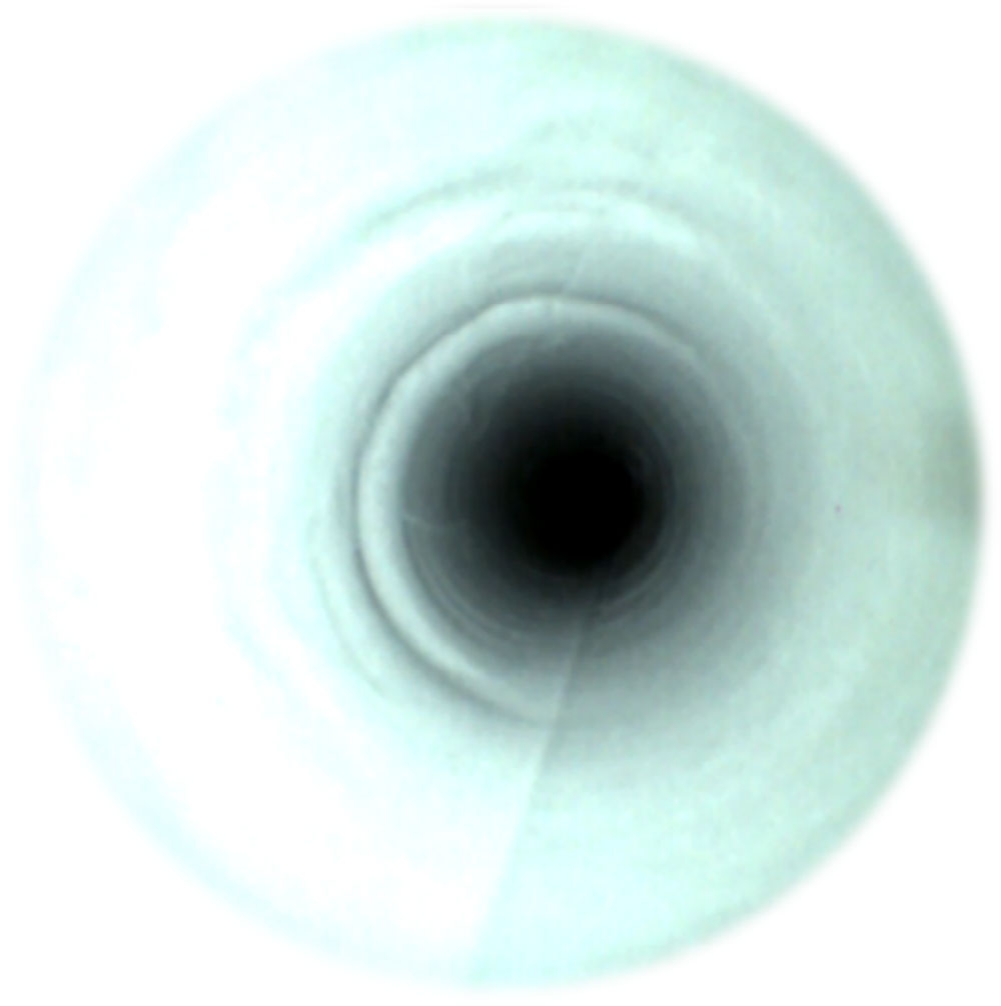
Narrowing in the endoscopic channel.

In contrast to the normal state (Figure 3), scratches were defined as scrape‐like marks, discoloration as clear color changes, and deformation as narrowing of the image. The survey results were compared with the target scope (Figure 4; Figure 5, 6, 7).

### Scope

The study used the EYEBOSCO inspection scope as the ultrafine‐diameter scope. The EYEBOSCO has an outer diameter of 2.0 mm, a length of 1.95 m, and is flexible and soft, making it ideal for observing the inside of a digestive endoscopic channel. This device facilitates observation inside the channel of a gastrointestinal endoscope.

The target endoscopes included upper gastrointestinal endoscopes (GIF‐H290: 5; GIF‐H290Z: 4; GIF‐Q260J: 3; GIF‐XZ1200: 2; GIF‐XP290N: 2; GIF‐H290EC: 1; EG‐580NW: 2; EG‐L590WR: 2; EG‐L600ZW: 2; EG‐L600ZW7: 2; Total: 25), colonoscopes (CF‐H260AI: 1; CF‐H290ECI: 1; CF‐H290I: 1; CF‐HQ290I: 1; CF‐Q260AI: 2; CF‐Q260DI: 1; CF‐XZ1200I: 1; PCF‐PQ260L: 1; PCF‐Q260AZI: 1; PCF‐Q260JI: 1; PCF‐Q260AI: 2; PCF‐H290TI: 1; PCF‐H290ZI: 2; PCF‐H290DI: 2; EC‐L600ZP: 4; EC‐L600ZP7: 1; EC‐L600XP7/L: 1; Total: 24), duodenoscopes (JF‐260V: 2; TJF‐260V: 1; Total: 3), echoendoscopes (GF‐UCT260: 1; GF‐UE260‐AL5: 1; EG‐740UT: 1; Total: 3), and double balloon endoscopes (EI‐580BT: 1; EN‐580T: 1; EN‐580XP: 1; Total: 3) manufactured by Olympus and Fujifilm, with forceps channels ranging from 2.0 to 4.2 mm. They were categorized into observation and therapeutic endoscopes according to their specified use (Table 1).

**TABLE 1:** Target endoscopes.

Aim	Observation, *N* = 24		Therapeutic, *N* = 34		*p*‐value
Company	O	F	O	F	
Upper gastrointestinal endoscope	14	8	3	0	0.527
Colonoscope	0	0	18	6	–
Duodenoscope	0	0	3	0	–
Echoendoscope	1	0	1	1	1.0
Double‐balloon endoscope	0	1	0	2	–
Total	15	9	25	9	0.402

Upper gastrointestinal endoscope	Company	Aim	Forceps channels ranging	From the date of purchase to test and inspection (months)	Time from last repair date to test inspection (months)
GIF‐H290	O	Observation use	2.8	55	7
GIF‐H290	O	Observation use	2.8	55	2
GIF‐H290	O	Observation use	2.8	7	No records of repair
GIF‐H290	O	Observation use	2.8	55	24
GIF‐H290	O	Observation use	2.8	55	36
GIF‐H290EC	O	Observation use	2.2	55	No records of repair
GIF‐H290Z	O	Observation use	2.8	79	26
GIF‐H290Z	O	Observation use	2.8	79	17
GIF‐H290Z	O	Observation use	2.8	76	No records of repair
GIF‐H290Z	O	Observation use	2.8	55	11
GIF‐Q260J	O	Treatment use	3.2	51	No records of repair
GIF‐Q260J	O	Treatment use	3.2	51	19
GIF‐Q260J	O	Treatment use	3.2	51	0
GIF‐XP290N	O	Observation use	2.2	51	7
GIF‐XP290N	O	Observation use	2.2	55	4
GIF‐XZ1200	O	Observation use	2.8	7	No records of repair
GIF‐XZ1200	O	Observation use	2.8	7	No records of repair
EG‐L580NW	F	Observation use	2.4	79	5
EG‐L580NW	F	Observation use	2.4	79	3
EG‐L590WR	F	Observation use	2.8	79	10
EG‐L590WR	F	Observation use	2.8	79	2
EG‐L600ZW	F	Observation use	2.8	79	13
EG‐L600ZW	F	Observation use	2.8	79	9
EG‐L600ZW7	F	Observation use	2.8	34	3
EG‐L600ZW7	F	Observation use	2.8	11	1

Colonoscope	Company	Aim	forceps channels ranging	From the date of purchase to test and inspection (months)	Time from last repair date to test inspection (months)
CF‐H260AI	O	Treatment use	3.7	127	No records of repair
CF‐H290ECI	O	Treatment use	3.2	55	No records of repair
CF‐H290I	O	Treatment use	3.2	55	36
CF‐HQ290I	O	Treatment use	3.7	114	30
CF‐Q260AI	O	Treatment use	3.2	234	25
CF‐Q260AI	O	Treatment use	3.2	234	8
CF‐Q260DI	O	Treatment use	3.2	112	49
CF‐XZ1200I	O	Treatment use	3.7	6	No records of repair
PCF‐H290DI	O	Treatment use	3.2	3	No records of repair
PCF‐H290DI	O	Treatment use	3.2	7	No records of repair
PCF‐H290TI	O	Treatment use	3.2	51	No records of repair
PCF‐H290ZI	O	Treatment use	3.2	55	No records of repair
PCF‐H290ZI	O	Treatment use	3.2	55	13
PCF‐PQ260L	O	Treatment use	2.8	79	11
PCF‐Q260AI	O	Treatment use	3.2	210	21
PCF‐Q260AI	O	Treatment use	3.2	234	3
PCF‐Q260AZI	O	Treatment use	3.2	127	No records of repair
PCF‐Q260JI	O	Treatment use	3.2	79	No records of repair
EC‐L600XP7/L	F	Treatment use	2.8	11	No records of repair
EC‐L600ZP	F	Treatment use	3.2	79	10
EC‐L600ZP	F	Treatment use	3.2	79	9
EC‐L600ZP	F	Treatment use	3.2	79	9
EC‐L600ZP	F	Treatment use	3.2	79	9
EC‐L600ZP7	F	Treatment use	3.2	58	12

Duodenoscope	Company	Aim	Forceps channels ranging	From the date of purchase to test and inspection (months)	Time from last repair date to test inspection (months)
JF‐260V	O	Treatment use	3.7	199	0
JF‐260V	O	Treatment use	3.7	55	3
TJF‐260V	O	Treatment use	4.2	76	No records of repair

Echoendoscope	Company	Aim	Forceps channels ranging	From the date of purchase to test and inspection (months)	Time from last repair date to test inspection (months)
GF‐UCT260	O	Treatment use	3.7	101	1
GF‐UE260‐AL5	O	Observation use	2.2	55	No records of repair
EG‐740UT	F	Treatment use	4.0	1	No records of repair

Double‐balloon endoscope	Company	Aim	Forceps channels ranging	From the date of purchase to test and inspection (months)	Time from last repair date to test inspection (months)
EI‐580BT	F	Treatment use	3.2	79	16
EN‐580T	F	Treatment use	3.2	79	15
EN‐580XP	F	Observation use	2.2	76	10

Abbreviations: F, Fujifilm; O, Olympus.

### Outcome measurements

The primary endpoints were the number of injuries in the observation and therapeutic endoscopes. The secondary endpoints were the type and number of injuries classified based on injury type and endoscope category.

### Statistical analyses

Categorical variables were compared between groups using Pearson's χ^2^ test. All statistical analyses were performed using IBM SPSS Statistics for Windows, version 26 (IBM Corp.). Statistical significance was considered at *p *< 0.05, and all tests were two‐sided.

## RESULTS

We surveyed 24 and 34 observation and therapeutic endoscopes, respectively, used at the Saitama Medical University Hospital. The observation set included 22 upper gastrointestinal endoscopes, one echoendoscope, and one double‐balloon endoscope. The procedure set included two upper gastrointestinal scopes, 24 colonoscopes, three duodenoscopes, two echoendoscopes, and two double‐balloon endoscopes (Table 1).

Overall, seven (29.2%) and 27 (79.4%) observation and therapeutic endoscopes, respectively, had scratches, discolorations, or deformations; injuries were more common in the therapeutic endoscopes (*p*‐value = 0.00006).

Six (25%) and 27 (79.4%) observation and therapeutic endoscopes, respectively, had scratches in the channel (*p* = 0.00001), indicating that the number of scratched therapeutic endoscopes was significantly higher than that of observation endoscopes.

Discoloration was found in two (8.3%) and 10 (29.4%) observation and therapeutic endoscopes, respectively (*p *= 0.052). One observation endoscope (4.2%) and two therapeutic endoscopes (5.9%) showed deformations in the channel (*p* = 0.776; Table 2).

**TABLE 2:** Breakdown of endoscopes with confirmed scratches, discoloration, or deformation.

	Observation, *N* = 24	Therapeutic, *N* = 34	*p*‐value
Total	7 (29.2%)	27 (79.4%)	0.00006
Scratches	6 (25%)	27 (79.4%)	0.00001
Discoloration	2 (8.3%)	10 (29.4%)	0.052
Deformation	1 (4.2%)	2 (5.9%)	0.776

The presence of damage (scratches, discoloration, and deformation) was also examined according to scope type. Among the 22 upper gastrointestinal scopes used for observation, six (16.7%) were found to be damaged. Three upper gastrointestinal scopes (100%) used for treatment were found to be damaged. Additionally, 23 (95.8%) of the 24 colonoscopes were damaged.

The observation echoendoscope was not damaged, although its therapeutic equivalent was damaged. The double‐balloon endoscope used for observation was damaged in one case.

Neither of the double‐balloon endoscopes used for treatment were damaged (Table 3).

**TABLE 3:** Comparison of scratches, discoloration, and deformation in endoscopic channels between observation and treatment use.

Aim	Observation, *N* = 24	Therapeutic, *N* = 34	*p*‐value
Upper gastrointestinal endoscope	6 (16.7%)	3 (100％)	0.0182
Colonoscope	0	23 (95.8%)	–
Duodenoscope	0	0	–
Echoendoscope	0	1 (50%)	–
Double‐balloon endoscope	1 (100%)	0	–
Total	7 (29.2%)	27 (79.4%)	0.00006

## DISCUSSION

Observation of the endoscopic channel and early detection of tract damage or discoloration may help prevent endoscopically transmitted infections. Among the various possible injuries to endoscopes, wounds are particularly problematic because bacteria can settle in them and cause infections. The endoscopic channel is easily damaged by the passage of forceps and other instruments. If organic matter adheres to the damaged area, we speculate that disinfection may be ineffective because its removal is difficult with standard cleaning methods. An Egyptian study showed that the reuse of biopsy forceps during colonoscopy increases the risk of Hepatitis C virus infection in patients.^6^

Damage to the scope after the procedure, particularly from scratches, may lead to an increase in bacterial growth. Composite infection rates have been reported at 0.2% after gastrointestinal endoscopic procedures, 0.8% after ERCP, 0.123% after non‐ERCP upper gastrointestinal endoscopic procedures, and 0.073% after lower gastrointestinal endoscopic procedures. *Pseudomonas aeruginosa* was the most prevalent organism, followed by other *Enterobacteriaceae* spp. and Gram‐positive cocci.^3^ Additionally, Pajkos et al. have previously confirmed biofilm formation in the endoscopic tract and reported that biofilm may contribute to endoscopic contamination.^5^

In this study, we anticipated that duodenoscopes, as well as upper and lower gastrointestinal endoscopes used for treatment and observation, would show predominant differences in damage. Using the EYEBOSCO enabled us to observe the inside of the endoscope channels and identify flaws and deformities that could not previously be detected. We found that damage was primarily observed in the scopes used for procedures. Most injuries were found on colonoscopes and were believed to be caused by the injection needles used for endoscopic mucosal resection and endoscopic submucosal dissection. The cause of the discolorations remains unknown but may change over time. External energy from dropping the scope may have caused the deformations. This may explain why no significant differences were observed in discoloration and deformation between the endoscopes used for observation and treatment in this study.

Scopes with confirmed flaws and deformities were significantly more likely to be in use for procedures. The use of instruments such as local injection needles for hemostasis and polyp/tumor resection may be responsible. Specifically, local injection needles can pass through the channel without being retracted into the sheath. Additionally, injury caused by rigid‐tipped instruments, such as clip forceps or grasping forceps, may also be a factor. The difference in the extent of channel damage between the observation and therapeutic endoscopes—specifically, the therapeutic endoscope, which passes through the lumen more frequently—demonstrates that the damage is to some degree related to the repeated passage of the device during the procedure. Therefore, particular attention should be paid to handling before and after inserting instruments, as damage to the channel is possible, especially near the tip or curvature of the scope.

Disposable endoscopes have recently gained attention owing to their comparable visibility and diagnostic capabilities to those of conventional endoscopes. Garbin et al. developed and validated a disposable endoscope that costs $357,^7^ whereas Bang et al. estimated that the break‐even cost of disposable duodenoscopes is over $1300 for low‐volume centers (less than 50 ERCPs/year) and over $800 for high‐volume centers (more than 150 ERCP/year), depending on the infection rate.^8^ Conversely, conventional endoscopes are estimated to incur an additional expenditure of $114 to $280 per use for reprocessing, along with the high initial cost of acquisition.^9^

A recent study suggested that disposable colonoscopes offer larger cost savings in facilities with low colonoscope use.^10^

Another issue with disposable endoscopes is their environmental impact. A recent study estimated the amount of non‐recyclable waste generated if disposable gastrointestinal endoscopes were universally adopted in the US compared to reprocessed endoscopes. The study estimated that reprocessed endoscopes generate approximately 532,918 m^3^ of waste yearly in the US, while disposable duodenoscopes and colonoscopes would generate an additional 100,682 m^3^ of waste yearly.^11^

Responding early to minute damage and deformation enables long‐term scope use and promotes the efficient use of resources, in line with the Sustainable Development Goals. The EYEBOSCO facilitates the observation of endoscopic channels, allowing for the examination of instrument effects on the channel and their relationship to scope failure.

The limitations of this study include the small number of cases in this pilot study and the fact that treatment time was not considered for each endoscope. The impact of these findings on infection rates remains unclear, as infection rates were not examined. Similarly, the effect of these findings on scope repair needs was not examined. At the very least, the endoscope manufacturer does not set any restrictions on use based on the degree of endoscope damage. Damage to the inside of the channel can only be confirmed by identifying leaks, using a cleaning brush, or noting any discomfort when inserting forceps. Therefore, if there is an air leak or any abnormalities with the scope's insertion, its use must be discontinued. Future studies should include larger samples and assess the impact of these factors on infection rates and repair demands.

In conclusion, observing the inside of the endoscopic channel with an ultrafine‐diameter scope enables the detection of microscopic damage and deformation. Periodic observation with an ultrafine‐diameter scope may promote the long‐term use of endoscopes.

## CONFLICT OF INTEREST STATEMENT

None.

## ETHICS STATEMENT

Ethical approval was not sought for the present study because this is not a study involving humans.

## Supporting information



Video EYEBOSCO.mp4

## Data Availability

All the data used to support the findings of this study are included in this article.
